# Oxidative Stress Sensing System for 8-OHdG Detection Based on Plasma Coupled Electrochemistry by Transparent ITO/AuNTAs/PtNPs Electrode

**DOI:** 10.3390/bios13060643

**Published:** 2023-06-12

**Authors:** Yongchang Bai, Shuang Li

**Affiliations:** Academy of Medical Engineering and Translational Medicine, Tianjin University, Tianjin 300072, China; baiyongchang@tju.edu.cn

**Keywords:** 8-hydroxydeoxyguanosine (8-OHdG), oxidative stress, indium tin oxide (ITO) electrode, integrated circuit system, point-of-care testing (POCT)

## Abstract

8-Hydroxydeoxyguanosine (8-OHdG) is the most widely used oxidative stress biomarker of the free radical-induced oxidative damage product of DNA, which may allow a premature assessment of various diseases. This paper designs a label-free, portable biosensor device to directly detect 8-OHdG by plasma-coupled electrochemistry on a transparent and conductive indium tin oxide (ITO) electrode. We reported a flexible printed ITO electrode made from particle-free silver and carbon inks. After inkjet printing, the working electrode was sequentially assembled by gold nanotriangles (AuNTAs) and platinum nanoparticles (PtNPs). This nanomaterial-modified portable biosensor showed excellent electrochemical performance for 8-OHdG detection from 10 μg/mL to 100 μg/mL by our self-developed constant voltage source integrated circuit system. This work demonstrated a portable biosensor for simultaneously integrating nanostructure, electroconductivity, and biocompatibility to construct advanced biosensors for oxidative damage biomarkers. The proposed nanomaterial-modified ITO-based electrochemical portable device was a potential biosensor to approach 8-OHdG point-of-care testing (POCT) in various biological fluid samples, such as saliva and urine samples.

## 1. Introduction

With the development of cancer research, although there are some clearly identified risk factors, such as smoking, drinking, obesity, and so on, aging is another factor that needs attention [1,2,3]. Therefore, as one’s age increases, the incidence rate of cancer increases sharply, which is due to the combination of a high risk of specific cancer and a low-efficiency cell repair mechanism [4,5,6]. In the context of the rapid development of global aging, if we can understand the processes of aging and how they affect the development of cancer and can achieve early detection and treatment, cancer mortality can be greatly reduced. Oxidative stress refers to an unbalanced and tending to oxidize state in the body, which is an important factor for aging and diseases, inducing destruction of the normal cell membrane and nuclear structure, protein structure changes, and chromosome aberrations [7,8,9]. In recent years, the application of biomarkers in patient status diagnosis and management has become a growing method, and the development of molecular biology has led to the continuous discovery of new circulating biomarkers [10,11]. At the same time, molecular epidemiological studies have also confirmed the relationship between oxidative stress and carcinogens [12,13].

Hydroxyl radicals may attack cellular membranes, proteins, nucleic acids, nuclear DNA, mitochondrial DNA, and RNA, during which 8-hydroxydeoxyguanosine (8-OHdG) forms abundantly and stablely [14]. At present, the most commonly used and promising biomarkers 8-hydroxydeoxyguanosine (8-OHdG) for oxidative stress have been investigated in medicine and toxicology [15,16]. It has been proposed that the content of 8-OHdG is the most studied biomarker of DNA damage-induced diseases, including cancer [17,18,19], neurodegenerative disorders [20,21], diabetes [22,23], cardiovascular [24,25], and infectious diseases [26]. Early detection is still the most crucial determinant for successful treatment and survival. Therefore, oxidative stress biomarker 8-OHdG assays carried out in wide screening programs by POCT are very useful for early diagnosis and assessment of susceptible populations. Research showed that 8-OHdG had been detected in various biological samples, such as plasma, urine, saliva, and tissue [27,28,29,30,31,32]. Among them, urinary biological samples were broadly used in preventive and occupational medicine due to their non-invasive sampling capabilities.

Various methods have been proposed to sense 8-OHdG in biological samples, such as gas chromatography-mass spectrometry (GC-MS), high performance liquid chromatography (HPLC), liquid chromatography-single mass spectrometry (HPLC-MS), enzyme-linked immunosorbent assay (ELISA), and resonance Rayleigh scattering (RRS) [33,34,35,36,37]. They showed high sensitivity in the 8-OhdG quantitative study due to the professional equipment and the multiple reaction monitoring mode, leading to a considerable reduction in the background ion-caused noise. However, the consumption of time, the requirement of sophisticated and laborious technologies, and the excessive handling of biological samples make them not conducive to on-site screening needs in special environments. While electrochemical and voltammetric techniques attract attention due to their portable, fast, easy-to-use, and low-cost analysis of biomolecules, drugs, and electroactive compounds in comparison to conventional analytical methods. Biosensors have been reported to achieve this through electrochemical or quartz crystal microbalance (QCM) detection technologies [38,39,40] by using the 8-OHdG active redox properties established in direct reading modes. However, the need for trace determination of 8-OHdG in complex matrices such as serum, urine, and saliva has not been met, which calls for more intense research. This is beneficial for establishing a POCT platform to transfer traditional diagnostic tests in the clinical laboratory setting to a near patient setting with timely diagnostic information, enabling better informed decisions regarding diagnosis.

In this work, we propose a sensitive oxidative stress biosensor by using the AuNTAs/PtNPs plasma-assembled ITO electrode for the detection of 8-OHdG. The sensing electrode adopted a multi-layer screen printing process and was made of special template-printed carbon paste and silver paste on the flexible and transparent ITO material substrate. AuNTAs were modified on the ITO electrode by electrostatic adsorption, and PtNPs were modified on the ITO/AuNTAs electrode by electrochemical reduction. Based on square wave voltammetry (SWV), PtNPs were prepared by reduction with chloroplatinic acid hexahydrate on the AuNTAs-modified electrode. Then a portable oxidative stress sensing system for electrochemical detection was developed with screen-printed sensing electrodes, printed circuit boards (PCBs), and PCs/smartphones. Finally, the layer-by-layer self-assembled electrodes and the portable sensing system were applied for 8-OHdG detection from 10 ng/mL to 100 μg/mL by differential pulse voltammetry (DPV). Here, the strategy of applying ITO/AuNTAs/PtNPs electrodes as sensing layers to achieve electrochemical analysis and enhance sensitivity is reported for the first time. Moreover, the fabricated oxidative stress sensing system exhibited good performance in POCT, demonstrating its promising application in biomarker monitoring.

## 2. Experimental Section

### 2.1. Chemicals and Materials

ITO-PET (resistance ≤ 6 ohm/sq) was obtained from HNXCKJ Co., Ltd. (Shenzhen, China). The hydrophobic layer was obtained from Neopro Co., Ltd. (Beijing, China). Silver ink was obtained from UVTM Co., Ltd. (Dongguan, China) (0.1 mg/mL Ag nanoparticles with dimethylbenzene as solvent). Carbon ink was obtained from JUJO Co., Ltd. (Tokyo, Japan) (5.0 wt% graphite powder with N-Methylpyrrolidone as solvent). Isopropanol (C_3_H_8_O), gold chloride trihydrate (HAuCl_4_·3H_2_O), chloroplatinic acid hexahydrate (H_2_PtCl_6_·6H_2_O), and sodium thiosulfate pentahydrate (Na_2_S_2_O_3_·5H_2_O) were obtained from Sigma-Aldrich (St. Louis, MO, USA). Phosphate buffered saline (PBS, pH = 7.4) was obtained from the Standard Information Network (China). 8-OHdG, potassium ferricyanide (K_3_Fe(CN)_6_), and potassium hexacyanoferrate (II) (K_4_Fe(CN)_6_) were obtained from Sigma–Aldrich (St. Louis, MO, USA).

### 2.2. Production of ITO Electrode

**Figure 1:**
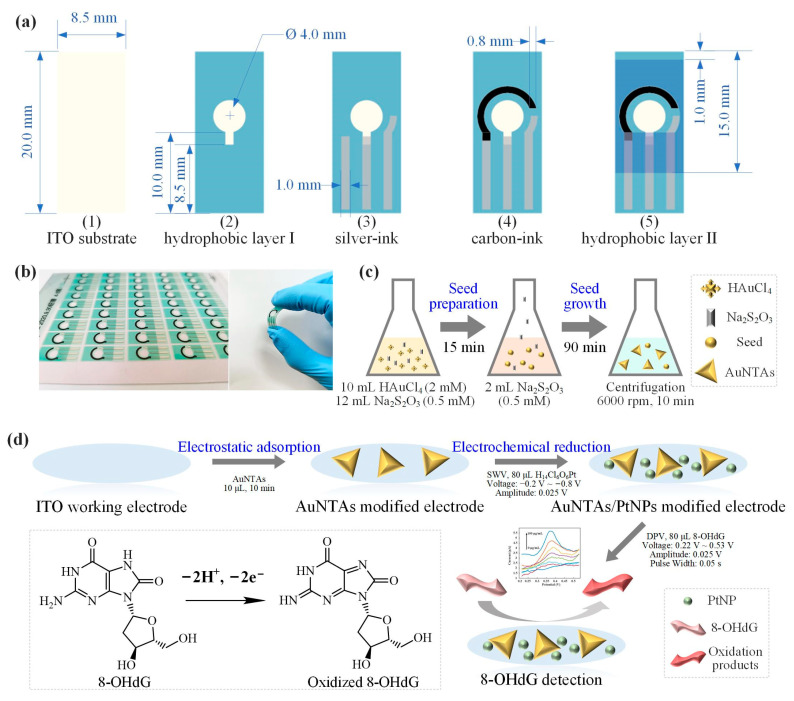
Plasma-assembled ITO electrode for 8-OHdG detection. (**a**) Fabrication process of ITO electrode. (**b**) Finished electrode product of batch printing. (**c**) Synthesis of AuNTAs. (**d**) 8-OHdG detection.

ITO electrodes were produced by an automatic screen-printing machine. Figure 1a shows the fabrication process of an ITO electrode: (1) Clean the ITO substrate with isopropanol. Each electrode was 20.0 mm long and 8.5 mm wide. (2) Cover hydrophobic layer-Ⅰ on the ITO substrate to expose the circular working electrode (WE) area and interface part. The diameter of WE was 4.0 mm. (3) Print three strips of silver ink with a width of 1.0 mm, and the right one was the reference electrode (RE). (4) Print arc-shaped carbon ink as a counter electrode (CE) with a width of 0.8 mm. (5) Cover hydrophobic layer-Ⅱ on the upper area of the ITO electrode to expose the detection area with CE and RE as boundaries. Figure 1b showed the finished electrode product of batch printing and the great flexibility of ITO electrode.

### 2.3. Synthesis of AuNTAs

AuNTAs were obtained by reducing hydrogen tetrachloroaurate (III) trihydrate with sodium thiosulfate pentahydrate, as in our previous work [41]. In seed preparation, 10 mL of HAuCl_4_·3H_2_O (2 mM) and 12 mL of Na_2_S_2_O_3_·5H_2_O (0.5 mM) were mixed in a 50 mL conical bottle and gently stirred for 15 min at room temperature (Figure 1c). Then 2 mL of Na_2_S_2_O_3_·5H_2_O (0.5 mM) was added to the solution for seed growth. After 90 min of stirring, the AuNTAs were formed. Finally, the AuNTAs solution was centrifugated at 6000 rpm for 10 min and stored at room temperature. Spectral analysis of AuNTAs was carried out by UV-vis-NIR (Lambda 750, Perkin Elmer, Waltham, MA, USA). Triangular features in the nanometer size of AuNTAs were observed by transmission electron microscopy (TEM, Tecnai G2 F20, Eindhoven, The Netherlands).

### 2.4. Plasma-Assembled ITO Electrode

Before the modification process, it was necessary to clean the electrode surface. At the start, deionized water was used to wash the electrode surface. Then 80 μL of PBS was dripped to the detection area of the electrode, using DPV to get the electrochemical cleaning effect. Scan from 0.15 to 0.35 V with a 25 mV amplitude, a 5 mV increment, a 50 ms pulse width, and a 500 ms pulse period. After about 6−8 cycles of scanning, a steady DPV response curve was observed. At last, deionized water was used to wash the electrode again. This cleaning process was very important to get a stable electrochemistry response during the further 8-OHdG detection process. After the cleaning process, the electrode should be dried with N_2_. Then 10 μL of AuNTAs solution was evenly dripped on the WE, and the whole electrode was put into the oven at 60 °C for 20 min (Figure 1d). Due to the electrostatic adsorption, AuNTAs were tightly adsorbed on the surface of WE so as to get an AuNTAs modified electrode. In electrochemical reduction, 80 μL of H_14_Cl_6_O_6_Pt was dripped to the detection area of the electrode, using SWV to electrodeposit PtNPs. Scan from −0.2 to −0.8V with 25 mV amplitude and 10 mV increments for 6 circles. Finally, an AuNTAs/PtNPs-modified electrode was prepared. CV was used to describe the electrochemical characterization of electrodes. A total of 80 μL of redox pairs (Fe^2+^/Fe^3+^) solution was scanned from −0.4 to 0.6 V with a scan rate of 50 mV/s. All electrochemical methods were carried out on a CHI660E electrochemical workstation (China). The morphological characteristics of the modified electrode were observed by scanning electron microscopy (SEM, NanoSEM 430, Rock Hill, SC, USA). Elemental analysis was carried out by energy dispersive spectroscopy (EDS, EMSA/MAS, London, UK).

### 2.5. 8-OHdG Detection

8-OHdG was dissolved in PBS to prepare standard solutions, whose concentrations ranged from 10 to 100,000 ng/mL. During the preparation, 5 min of shaking were needed to ensure full dissolution. DPV was used for 8-OHdG detection, scanning from 0.22 to 0.53 V with a 25 mV amplitude, a 5 mV increment, a 50 ms pulse width, and a 500 ms pulse period. Before each scan, 80 μL of the standard solution was added to the detection area of the ITO/AuNTAs/PtNPs electrode, and the 8-OHdG in the solution would be oxidized gradually with the variation of potential and form a peak current. After each scan, deionized water was used to clean the electrodes. In order to obtain reliable and reproducible results, three electrodes need to be prepared and used to scan solutions of each concentration (10 ng/mL, 100 ng/mL, 1 μg/mL, 5 μg/mL, 10 μg/mL, 50 μg/mL, 100 μg/mL). All detections were performed at room temperature. In data processing, mathematical analysis methods were used to calculate the peak current of standard solutions with different concentrations and observe the correlation between concentration and peak current.

## 3. Results and Discussion

### 3.1. Oxidative Stress Sensing System

**Figure 2:**
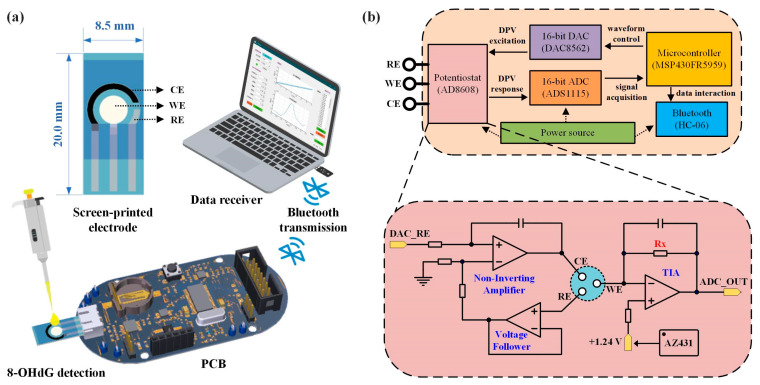
Oxidative stress sensing system. (**a**) The system consists of screen-printed sensing electrodes, PCBs, and PCs/smartphones. (**b**) PCB circuit composition. (CE: counter electrode; WE: working electrode; RE: reference electrode).

We have developed a portable oxidative stress sensing system for the electrochemical detection of 8-OHdG. The system consists of screen-printed sensing electrodes, printed circuit boards (PCBs), PCs, or smartphones (Figure 2a). The sensing electrode adopted a multi-layer screen printing process and was made of special template-printed carbon paste and silver paste on the flexible and transparent ITO material substrate. It contained the WE of ITO material, the RE of Ag/AgCl material, and the CE of carbon material. The size of a single sensing electrode was 8.5 × 20.0 mm, and the circular detection area was about 0.5 cm^2^. The small size of the electrode ensured that the detection of 8-OHdG concentration would be completed only by dripping about 50 μL of test solution into the detection area. The sensing electrode was connected to the three-electrode interface of the PCB head, and the PCB controlled the electrode to output and collect electrical signals in electrochemical detection. The collected data was transmitted to a PC or smartphone through Bluetooth and finally processed to obtain relevant concentration information. This portable electrochemical detection device had the characteristics of being fast, accurate, and low-power consumption and was a relatively common platform in electrochemical detection. It can realize a variety of electrochemical detection methods, such as CV, DPV, SWV, etc. It can carry different sensing electrodes to detect different biomarkers.

As shown in Figure 2b, the PCB was composed of a power supply, microcontroller, potentiostat module, digital-to-analog conversion module (DAC), analog-to-digital conversion module (ADC), Bluetooth module, etc. The microcontroller controlled the DAC to generate DPV voltage excitation and applied it to the sensor electrode through the potentiostat module. At the same time, the ADC collected the current signal generated in the chemical reaction (DPV response) and sent it to the upper computer through Bluetooth transmission for data processing and result display. The core circuit module was the potentiostat module, which was connected to the DAC and ADC inside the PCB and directly connected to the sensing electrode through the RE/WECE interface. Figure 2b shows the schematic diagram of the relevant circuit design. The potentiostat consisted of an in-phase amplifier, a voltage follower, a transimpedance amplifier (TIA), and a reference source. WE and RE formed a voltage control loop, using the voltage feedback effect of the amplifier to construct an in-phase amplifier and a voltage follower to accurately control the voltage. The voltage at the RE end was controlled by the DAC (DAC_RE), and the bias voltage at the WE end was controlled by the reference source chip AZ431 and ensured to be stable at 1.24V left to right. This way, the voltage difference between RE and WE can be adjusted within a certain positive and negative range. WE and CE formed a current loop and converted current to voltage through TIA, thereby outputting to the ADC for signal acquisition (ADC_OUT). Adjusting the size of the resistor Rx can change the output voltage, thereby adjusting the detection sensitivity.

### 3.2. Characteristics of ITO/AuNTAs/PtNPs Electrode

**Figure 3:**
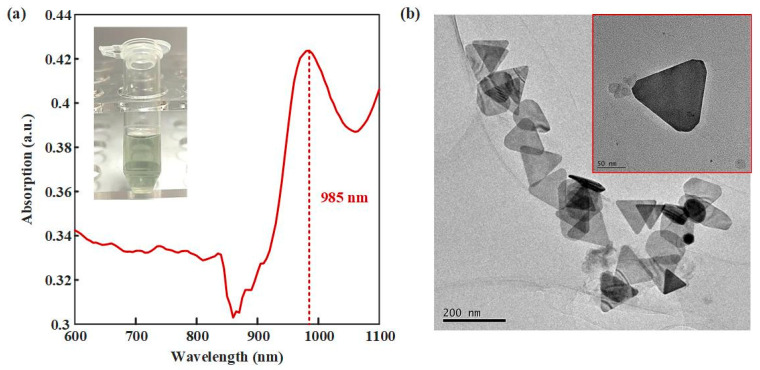
Characteristics of AuNTAs. (**a**) UV-vis-NIR spectrum of AuNTAs. (**b**) TEM of AuNTAs.

**Figure 4:**
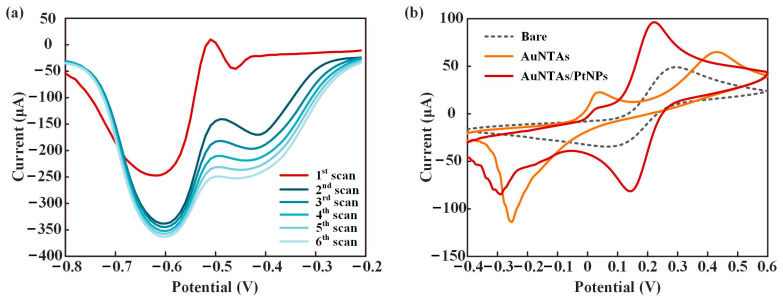
Characteristics of ITO/AuNTAs/PtNPs electrode. (**a**) SWV curves of PtNPs electrodeposition. (**b**) CV curves of bare electrode, AuNTAs modified electrode and AuNTAs/ PtNPs modified electrode.

UV-vis-NIR and TEM were used to verify the synthesis effect of AuNTAs. As shown in Figure 3a, an obvious absorption peak appeared at the 985 nm wavelength, which proved the success of the synthesized AuNTAs. Figure 3b shows the regular triangular shapes of AuNTAs under the TEM. The average particle size of AuNTAs was about 130 nm. Figure 4a shows the SWV response curves for PtNP electrodeposition. A great gap appeared in the first two scans and gradually decreased in the next four scans, which indicated that PtNPs were ultimately deposited on the WE. Figure 4b shows the CV response curves of a bare electrode, an AuNTAs modified electrode, and an AuNTAs/ PtNPs modified electrode. Compared with a bare electrode (Ep = 0.294 V, ip = 4.907 × 10^–5^ A), AuNTA’s modified electrode showed that the oxidation peak current rose and the peak position shifted to the right (Ep = 0.431 V, ip = 6.495 × 10^–5^ A). It indicated that AuNTAs had a specific enhancing effect on ITO electrodes because of their nano triangular features. The AuNTAs/PtNPs modified electrode showed a great current enhancement characteristic (Ep = 0.222V, ip = 9.636 × 10^–5^ A) as a result of the formation of arranged PtNPs, which improved the dielectric properties on the sensor surface. In addition, a phenomenon that could be observed was that the position of the oxidation peak had shifted back to the left, which may be related to the fully covered PtNPs on the WE surface.

**Figure 5:**
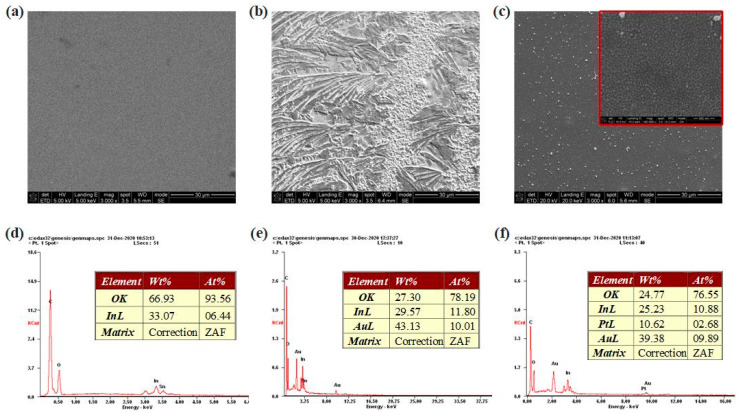
Morphology and elemental analysis of electrodes. SEM images of (**a**) bare electrode, (**b**) AuNTAs modified electrode, (**c**) AuNTAs/PtNPs modified electrode. EDS analyses of (**d**) bare electrode, (**e**) AuNTAs modified electrode, (**f**) AuNTAs/PtNPs modified electrode.

SEM images and EDS analyses of bare electrodes, AuNTAs-modified electrodes, and AuNTAs/PtNPs-modified electrodes are shown in Figure 5a–c. Bare ITO electrodes showed a clean and smooth surface, while AuNTA-modified electrodes had an apparently rough surface. Under the excitation of negative potential, PtNPs were evenly and densely tiled on the WE, which increased the contact area between test sample and electrode so as to improve the sensitivity. Figure 5d–f showed the elemental type and content changes in electrode modification through EDS. The elements of the bare ITO electrode mainly contained O and In, and the weight percentages were 66.93 and 33.07%, respectively. When AuNTAs were modified, Au became the main element of the electrode, whose weight percentage reached 43.13% and atomic percentage reached 10.01%. When PtNPs were electrodeposited on AuNTA’s modified electrode, the Pt element in the electrode showed up with a 10.62% weight percentage and a 2.68% atomic percentage. These results strongly proved the preparation success of the ITO/AuNTAs/PtNPs electrode.

### 3.3. 8-OhdG Detection

**Figure 6:**
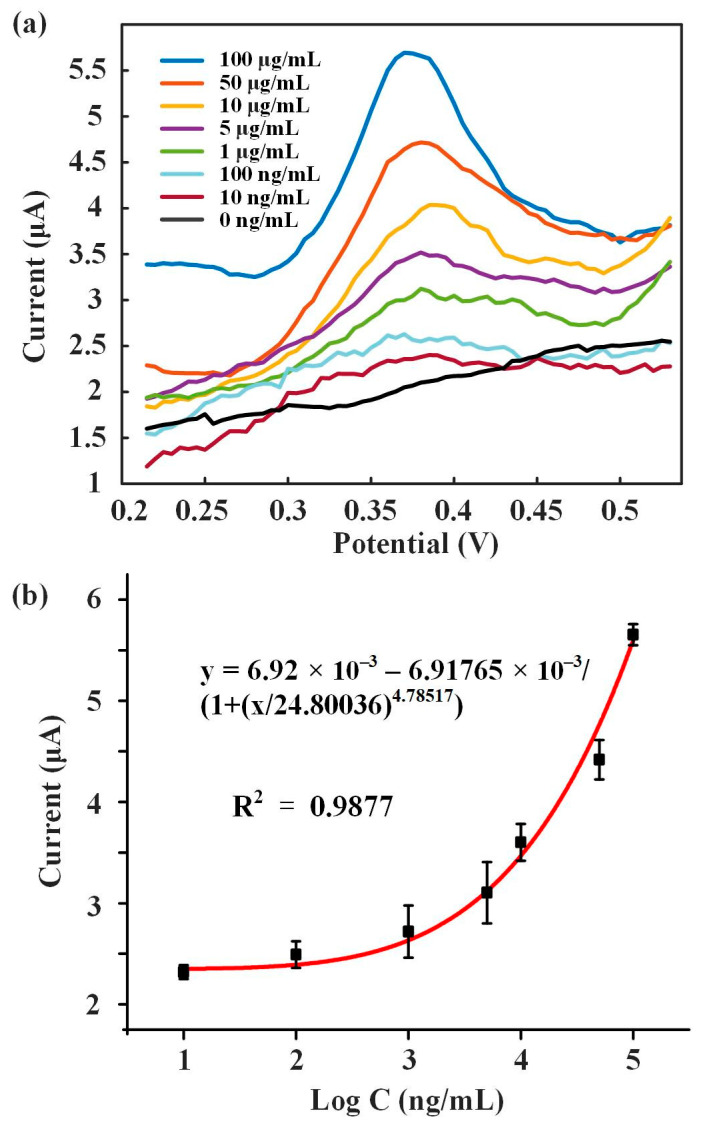
8-OhdG detection. (**a**) DPV analysis of 8-OhdG from 10 to 100 μg/mL. (**b**) Fitting curve of the concentration of 8-OHdG and peak current.

Different concentrations of 8-OHdG solutions (10 ng/mL, 100 ng/mL, 1 μg/mL, 5 μg/mL, 10 μg/mL, 50 μg/mL, 100 μg/mL) were detected. Figure 6a shows the DPV response curves of these solutions. 8-OHdG was fully oxidized at around 0.38 V and formed the peak current. As the concentration of 8-OHdG increased, the peak current also improved. Figure 6b showed the fitting curve of the concentration of 8-OHdG and peak current, between which the logarithmic fitting relationship was y = 6.92 × 10^–3^ − 6.91765 × 10^–3^/(1 + (x/24.80036)^4.78517^) with a R^2^ of 0.9877. The results showed that this method was feasible for detecting 8−OHdG. A comparison with reported 8−OHdG investigations is presented in Table 1, and it is found that our sensor has a wide detection range of 4 orders of magnitude and a low detection limit.

**Figure 7:**
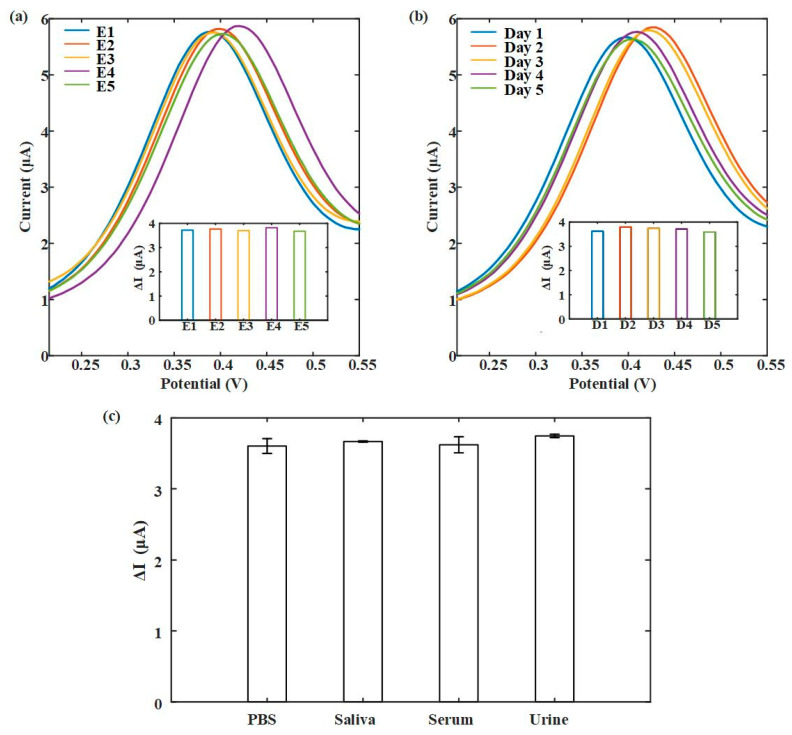
Characterization test. (**a**) Reproducibility test. (**b**) Stability test. (**c**) Saliva, serum, and urine samples test. (8-OhdG detection at 100 μg/mL).

In order to provide information about the reproducibility, stability, and sample testing of the biosensor developed in this work. Characterization test results are shown in Figure 7. Five sensors were used for 8-OhdG detection at 100 μg/mL, and the results showed that these sensors had similar DPV response signals (Figure 7a). This indicates that our sensor has good reproducibility. Furthermore, after five consecutive days of testing, the sensor still maintained a stable DPV signal output (Figure 7b). Due to the previous use of PBS to explore the sensibility of the test, in the end, we evaluated the device using relevant samples such as saliva, serum, and urine (classical samples to test for oxidative stress). Figure 7c showed that the current changes (ΔI) were consistent with PBS when detecting the same concentration of 8-OhdG in different sample environments. This indicates that our sensor has a sensitive response to 8-OhdG and exhibits excellent potential in actual sample testing.

## 4. Conclusions

In this study, a plasma-coupled electrochemistry electrode was proposed by transparent ITO/AuNTAs/PtNPs for oxidative stress sensing of 8-OHdG. ITO electrodes were prepared by layer-by-layer screen printing. A plasma-assembled ITO electrode was functionalized by electrostatic adsorption of AuNTAs and electrochemical reduction of PtNPs, which achieved high-precision sensing of 8-OHdG. Then a portable electrochemical detection device with a power supply, microcontroller, potentiostat module, DAC, ADC, and Bluetooth module was designed for fast, accurate, and low power consumption. The prepared oxidative stress sensing system exhibited good linearity in the concentration range of 8-OHdG from 10 ng/mL to 100,000 ng/mL. We also evaluated the device using relevant samples such as saliva, serum, and urine. Therefore, it is expected to become a common platform in electrochemical detection, can realize CV, DPV, and SWV with specific signal stimuli, and shows great potential for further development in POCT.

## Figures and Tables

**Table 1:** A comparison with reported 8-OHdG investigations. (MWCNT: multiwalled carbon nanotubes; SWCNT: single-wall carbon nanotubes; PEI: polyethylenimine; ASV: anodic stripping voltammetry; ErGO: electrochemically reduced graphene oxide; MIP: molecular imprinted polymers.).

Technique	Linear Range	Detection Limit	Real Sample	Reference
MWCNT/GCE (CV)	0.08–5 µM	9 nM	No	[42]
SWCNT-Nafion/GCE (DPV)	0.03–1.25 µM	8 nM	No	[43]
CNT-PEI/GCE (ASV)	0.5–30 µM	100 nM	No	[44]
MWCNT/ErGO/GCE (SWV)	3–75 µM	35 nM	Yes	[40]
MIP Sensor (SWV)	0.020–3 μM	3 nM	Yes	[45]
ITO/AuNTAs/PtNPs (DPV)	10–100,000 ng/mL	10 ng/mL	Yes	Present work

## Data Availability

Not applicable.
